# Giant left myxoma with mitral valve obstruction

**DOI:** 10.1002/ccr3.6406

**Published:** 2022-10-03

**Authors:** Valdano Manuel, Glória Pedro, Joaquim Gouveia, Belmira Luís, Sandra Armando, Damião António, Carlos Alberto Masseca, Sílvia Lutucuta

**Affiliations:** ^1^ Complexo Hospitalar de Doenças Cardio‐Pumonares Cardeal Dom Alexandre do Nascimento Luanda Angola; ^2^ Service of Internal Medicine Hospital do Prenda Luanda Angola

**Keywords:** mitral valve obstruction, myxoma, tumor

## Abstract

We present a case of a patient admitted with acute pulmonary edema. An echocardiogram showed a giant myxoma of the left atrium causing mitral valve obstruction. The patient underwent urgent cardiac surgery for tumor resection. There were no postoperative complications, and the follow‐up was uneventful.

## Graphical abstract

This case highlights that left atrium polypoid giant myxoma can cause acute pulmonary edema that can potentially be misdiagnosed as other cardiopulmonary diseases. Transthoracic echocardiogram is the imaging method of choice for the diagnosis of myxoma. Urgent cardiac surgery should be considered for treatment, as it is safe and has excellent results.

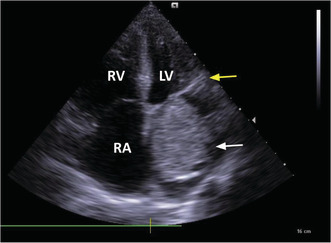

## 1 CASE

**FIGURE 1:**
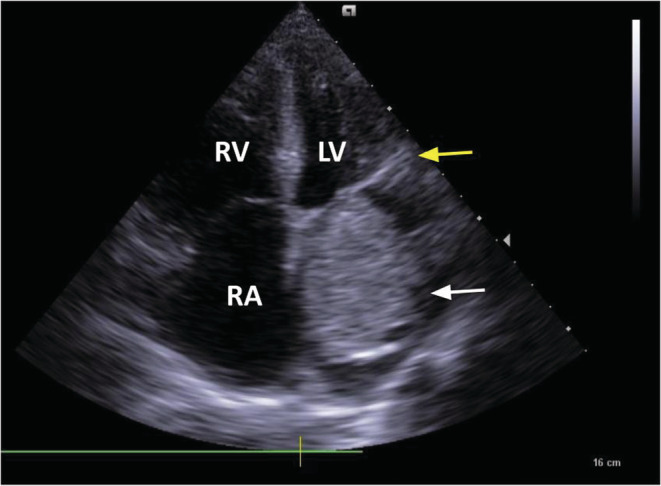
Transthoracic echocardiogram showing a giant mass (white arrow) in the left atrium obstructing the mitral valve (yellow arrow) that is suggestive of myxoma

A 66‐year‐old female was admitted to the hospital emergency department with acute pulmonary edema. Past medical history revealed previous hospitalization with respiratory symptoms, though these were not well clarified. In order to determine etiology, a transthoracic echocardiogram was performed. This showed a giant myxoma of the left atrium, causing obstruction of the mitral valve (Figure 1).

**FIGURE 2:**
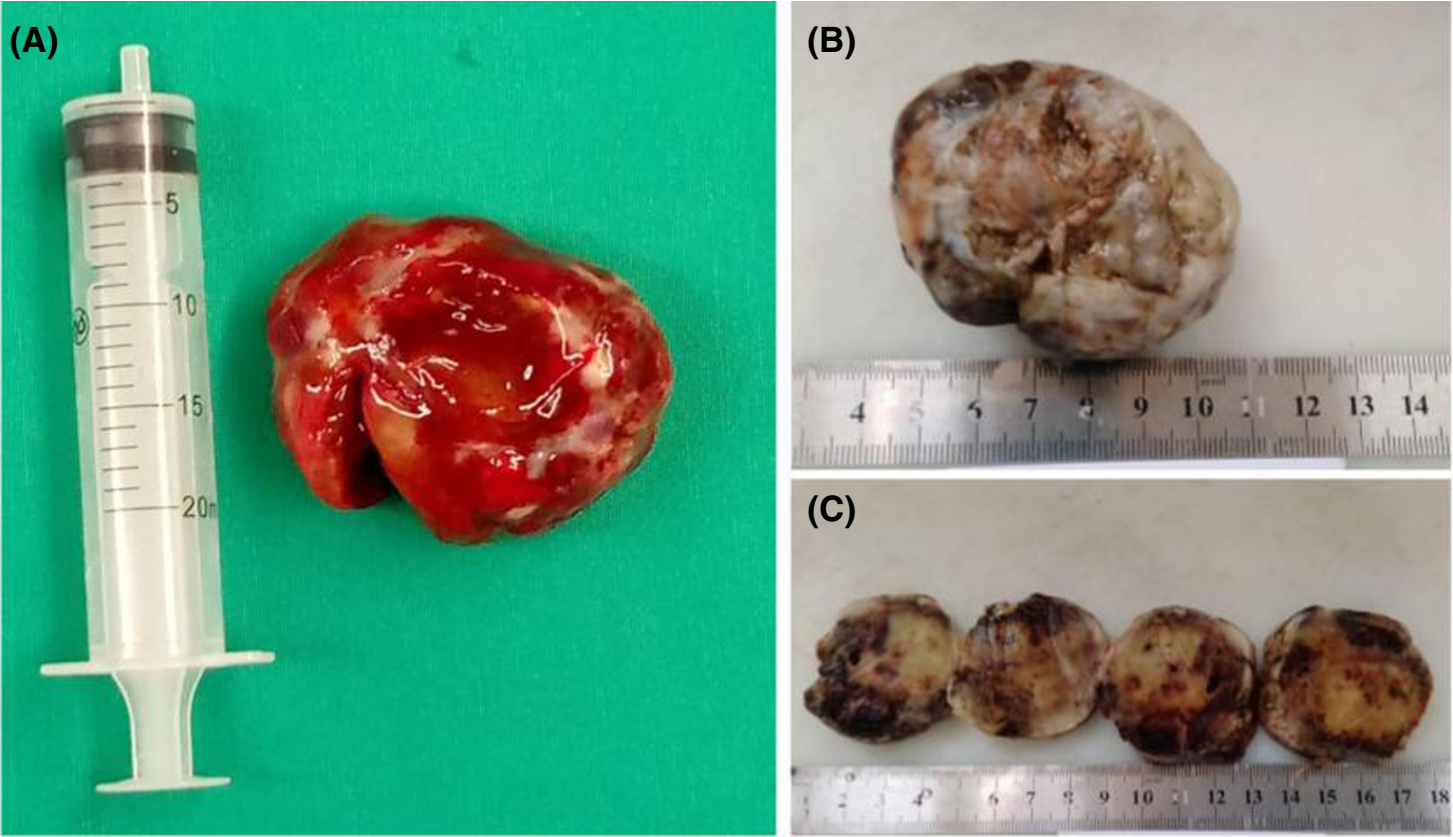
(A) A giant myxoma measuring more than 10 cm at its largest diameter. (B, C) Samples in preparation for histological analysis

**FIGURE 3:**
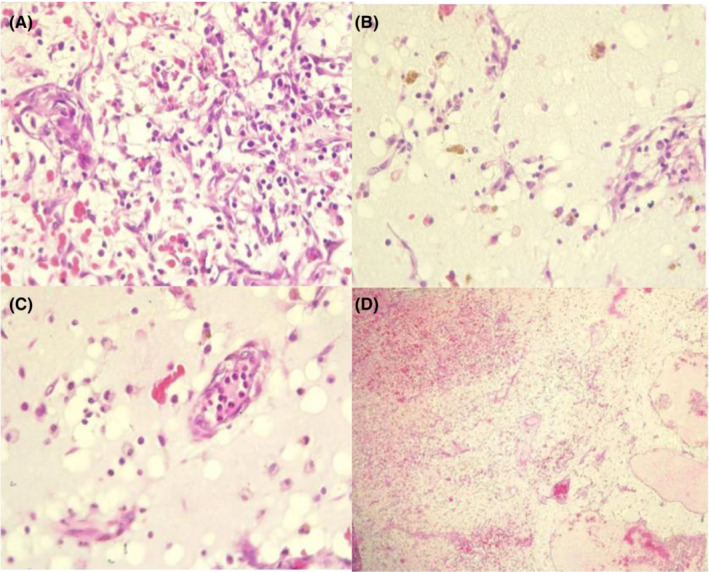
Atrial myxoma. (A) Diversity in the morphology of myxoma cells. Myxoma cells are isolated and arranged in a cord‐like structure scattered within the myxoid stroma. (B) Arrangement of polygonal myxoid cells and hemosiderin‐laden macrophages in a myxoid stroma. (C) Myxoma cells in small perivascular nests. (D) Myxoid cells adjacent to the area of hemorrhage, surrounded by myxoid stroma containing a large number of lymphocytes

The patient underwent urgent cardiac full sternotomy under cardiopulmonary bypass and cardiac arrest with antegrade St Thomas cardioplegic solution. Following a transverse left atrium incision, the large myxoma with a pedicle was observed in the interatrial septum above the mitral valve. The tumor was successfully resected (Figure 2), and the intraoperative course was uneventful. Histopathological examination confirmed the diagnosis of myxoma (Figure 3). The patient was followed up at three‐month post‐surgery and remains asymptomatic.

Cardiac tumors are rare, and metastasis is more common than that in primary tumors.^1^ Myxoma is a benign and the most frequent cardiac primary tumor. Depending on the size and location, the clinical manifestations vary greatly from asymptomatic to multi‐systemic manifestations.^2^, ^3^ Giant myxoma is defined as a tumor greater than 5 cm. When the myxoma of the left atrium is large, there is a risk of mitral valve obstruction, and consequently, the clinical manifestation can be acute pulmonary edema, as in the present case.

## AUTHOR CONTRIBUTIONS

Valdano Manuel performed the surgery, idealized and wrote, and revised it critically. Glória Pedro made the diagnosis and follow‐up of the patient as well as critically revised the manuscript. Joaquim Gouveia performed the surgery and treated the images. Sandra Armando performed the histological analysis and granting of images. Belmira Luís was the anesthesiologist and critically revised the manuscript. Damião António, Carlos Alberto Masseca, and Sílvia Lutucuta revised it critically and approved the final version.

## FUNDING INFORMATION

None.

## CONFLICT OF INTEREST

None.

## CONSENT

Written informed consent was obtained from the patient to publish this report in accordance with the journal's patient consent policy.

## Data Availability

Data available on request from the authors.
